# An experimental and theoretical NMR study of NH-benzimidazoles in solution and in the solid state: proton transfer and tautomerism

**DOI:** 10.3762/bjoc.10.168

**Published:** 2014-07-16

**Authors:** Carla I Nieto, Pilar Cabildo, M Ángeles García, Rosa M Claramunt, Ibon Alkorta, José Elguero

**Affiliations:** 1 Departamento de Química Orgánica y Bio-Orgánica, Facultad de Ciencias, UNED Senda del Rey 9, E-28040 Madrid, Spain; 2 Instituto de Química Médica, CSIC, Juan de la Cierva, 3, E-28006 Madrid, Spain

**Keywords:** CPMAS, DNMR, GIAO, proton transfer, tautomerism

## Abstract

This paper reports the ^1^H, ^13^C and ^15^N NMR experimental study of five benzimidazoles in solution and in the solid state (^13^C and ^15^N CPMAS NMR) as well as the theoretically calculated (GIAO/DFT) chemical shifts. We have assigned unambiguously the "tautomeric positions" (C3a/C7a, C4/C7 and C5/C6) of NH-benzimidazoles that, in some solvents and in the solid state, appear different (blocked tautomerism). In the case of 1*H*-benzimidazole itself we have measured the prototropic rate in HMPA-*d*_18_.

## Introduction

Of almost any class of heterocycles it can be said that they have relevant biological and medicinal chemistry properties, because, for instance, over 80% of top small molecule drugs by US retail sales in 2010 contain at least one heterocyclic fragment in their structures [1]. Benzimidazoles besides being the skeleton of many relevant drugs (fungicides, anthelmintics, antiulcerative, antiviral,…) [2–3] are also part of some natural products (the most prominent benzimidazole compound in nature is *N*-ribosyl-5,6-dimethylbenzimidazole, which serves as an axial ligand for cobalt in vitamin B_12_) and have interesting ferroelectric properties [4]. Particularly relevant for the present work is their proton conducting abilities, based of the 1,3-N–H···N hydrogen bonds, not only in benzimidazole polymers but in molecular compounds [5–6].

Degenerated tautomerism (autotrope) [7–8] simultaneously simplifies and complicates the NMR spectra of molecules in solution to the point that the assignment of some signals that become magnetically equivalent (isochronous) [9–10] by fast proton exchange has been much neglected. With the advent of solid-state NMR spectroscopy and the suppression of prototropic tautomerism, the assignment problem arises anew. In recent years, the use of very pure NMR solvents, particularly DMSO-*d*_6_, and highfield instruments has lead to obtain solution spectra where the prototropy has been considerably slowed down.

**Figure 1:**
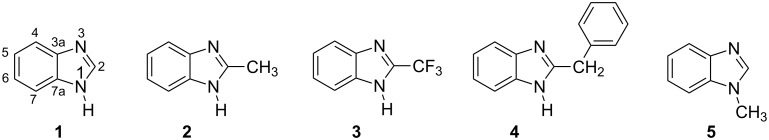
The five studied compounds.

We present in this paper a study of four *N*-unsubstituted 1*H*-benzimidazoles (Figure 1) including 2-methyl-1*H*-benzimidazole (**2**) that shows in the solid state ferroelectric switching in two dimensions due to its pseudo-tetragonal crystal symmetry [4], and 2-benzyl-1*H*-benzimidazole (**4**) a vessel-dilating and spasm-reducing agent known as dibazol or bendazole [3], where the tautomerism has been blocked resulting in the concomitant problem of assignment of some signals. We have selected 1-methyl-1*H*-benzimidazole (**5**) as the simplest benzimidazole without tautomerism.

The use of theoretically calculated chemical shifts has been decisive to solve this problem. We have used from 2001 [at the 6-31+G(d) level] [11] and then, from 2007, at the 6-311++G(d,p) level [12], a statistical approach that consists in comparing GIAO calculated absolute shieldings (σ, ppm) with experimental chemical shifts (δ) determined either in solution or in the solid state:

δ^1^H = 31.0 – 0.97 σ^1^H [13]

δ^13^C = 175.7 – 0.963 σ^13^C [12]

δ^15^N = –154.0 – 0.874 σ^15^N [12]

These equations give excellent results except for atoms (generally, carbon) linked to halogen atoms (I > Br > Cl >> F) [11,14] where relativistic corrections are necessary [15].

## Results and Discussion

The experimental data are all original although averaged values of all nuclei have been reported for the three NH-benzimidazoles **1**, **2** and **4** [16–21]; besides ^13^C and ^15^N NMR chemical shifts of **5** have been published [22–23].

**Figure 2:**
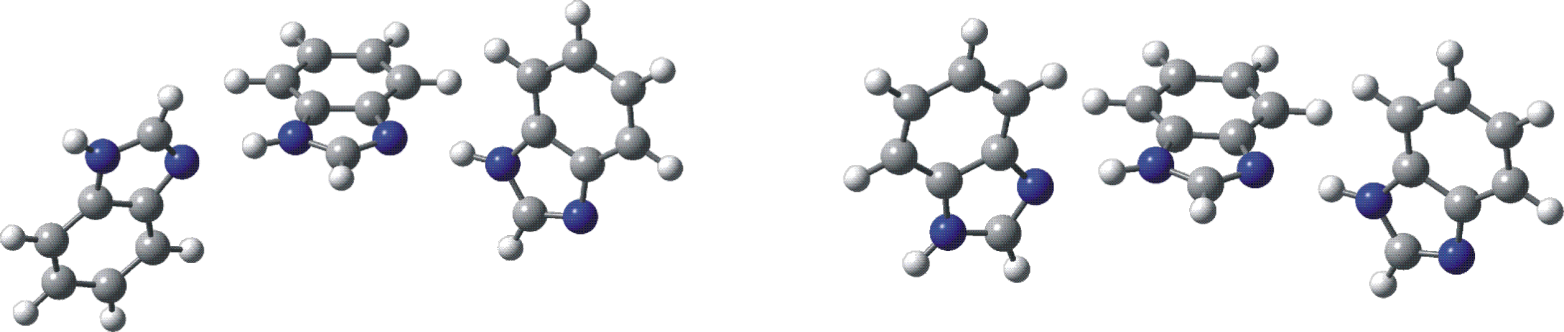
Trimers A (left) and B (right) of **1**.

Three kinds of calculations have been done: isolated molecules (gas phase), continuous model solvated molecules in DMSO, and hydrogen-bonded trimers for **1**, to simulate the crystal [24]. The central benzimidazole is N–H···N hydrogen bonded to two other benzimidazoles like in the crystal chain (catemer). Two trimers, A and B, that differ in the conformation of the first benzimidazole were calculated for this compound, but the differences in energy are very small, less than 0.1 kJ·mol^−1^ (Figure 2).

**Figure 3:**
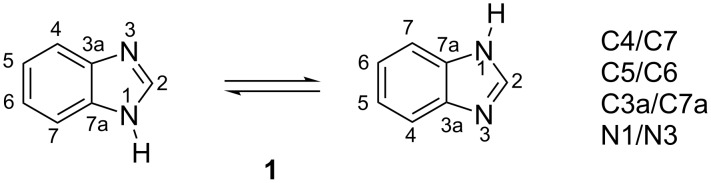
The tautomerism of 1*H*-benzimidazole.

First, we will discuss the six carbon atoms of the benzene ring of benzimidazole (3a, 4, 5, 6, 7, 7a) and then the remaining atoms (N1, C2, N3 and those of the substituent). In NH-benzimidazoles, when prototropic tautomerism occurs (Figure 3), the signals of the benzimidazole carbons in groups of two coalesce in an average signal (the same happens with the N atoms but the chemical shifts are so different that there are no problems of assignment). Actually, this was a very common occurrence, but in the series of compounds of Table 1, only for benzimidazole itself and for **3**, average signals were observed in DMSO-*d*_6_, a solvent known to slow down the prototropic exchanges. For these two compounds, we recorded the spectra in HMPA-*d*_18_, which is better for this purpose [25] observing the signals of compounds where the prototropic exchange is blocked.

**Table 1:** Calculated and experimental ^1^H and ^13^C chemical shifts of the benzene part.

Compound	^1^H calcd gas	^1^H calcd DMSO	^1^H exp. HMPA-*d*_18_ 273 K	^13^C calcd gas	^13^C calcd DMSO	^13^C exp. HMPA-*d*_18_ 273 K	^13^C exp. CPMAS

**1**							
4	7.81	7.79	7.54	121.67	119.58	118.7	119.4
5	7.20	7.34	7.11	121.68	121.79	120.1	123.0
6	7.24	7.41	7.11	122.88	123.04	121.2	123.8
7	7.32	7.67	7.42	108.23	110.99	110.9	112.9
3a	–	–	–	144.67	144.22	143.4	143.1
7a	–	–	–	132.70	133.54	133.7	136.4
**1** tri A							
4	7.89	7.93	7.54	119.85	118.28	118.7	119.4
5	7.26	7.39	7.11	122.10	121.91	120.1	123.0
6	7.28	7.39	7.11	122.93	122.73	121.2	123.8
7	7.49	7.77	7.42	110.00	111.88	110.9	112.9
3a	–	–	–	143.85	143.62	143.4	143.1
7a	–	–	–	134.13	134.76	133.7	136.4
**1** tri B							
4	7.91	7.90	7.54	119.99	118.42	118.7	119.4
5	7.28	7.38	7.11	122.02	121.87	120.1	123.0
6	7.26	7.39	7.11	122.72	122.68	121.2	123.8
7	7.48	7.75	7.42	110.18	112.00	110.9	112.9
3a	–	–	–	143.84	143.35	143.4	143.1
7a	–	–	–	134.04	134.62	133.7	136.4

Compound	^1^H calcd gas average	^1^H calcd DMSO average	^1^H exp. DMSO-*d*_6_ average	^13^C calcd gas average	^13^C calcd DMSO average	^13^C exp. DMSO-*d*_6_ average	^13^C exp. CPMAS

**1**							
4/7	7.695	7.825	7.58	115.08	115.21	115.3	
5/6	7.27	7.385	7.17	122.37	122.28	121.7	
3a/7a			–	138.94	138.98	138.4	
NH			12.44				
**2**							
4	7.70	7.68	7.47	120.78	118.54	117.8	117.4
5	7.11	7.23	7.11	121.22	121.09	120.7	121.7
6	7.06	7.21	7.10	121.91	121.86	121.2	121.7
7	7.21	7.54	7.40	107.79	110.32	110.5	111.6
3a	–	–	–	145.08	144.60	143.5	142.9
7a	–	–	–	135.53	136.25	134.3	134.7
NH			12.14				

**3**	^1^H calcd gas	^1^H calcd DMSO	^1^H exp. HMPA-*d*_18_ 273 K	^13^C calcd gas	^13^C calcd DMSO	^13^C exp. HMPA-*d*_18_ 273 K	^13^C exp. CPMAS

4	7.87	7.88	7.72	122.65	120.89	120.8	114.7 118.0
5	7.43	7.59	7.37	124.08	124.84	123.2	120.3 122.1
6	7.37	7.57	7.42	125.89	126.68	125.1	128.1 130.7
7	7.31	7.67	7.58	109.07	112.00	112.9	108.2 109.3
3a	–	–	–	144.54	143.87	142.9	
7a	–	–	–	132.27	133.24	135.1	
NH			15.05				

**4**	^1^H calcd gas	^1^H calcd DMSO	^1^H exp. DMSO-*d*_6_	^13^C calcd gas	^13^C calcd DMSO	^13^C exp. DMSO-*d*_6_	^13^C exp. CPMAS

4	7.74	7.70	7.52	120.46	118.51	118.3	120.2
5	7.27	7.38	7.10	121.91	122.22	120.9	123.0
6	7.12	7.28	7.11	122.64	122.79	121.6	124.8
7	6.95	7.32	7.40	107.24	109.79	110.9	113.5
3a	–	–	–	144.14	143.43	143.4	143.0
7a	–	–	–	134.27	135.43	134.3	135.2
NH			12.28				

**5**	^1^H calcd gas	^1^H calcd DMSO	^1^H exp. DMSO-*d*_6_	^13^C calcd gas	^13^C calcd DMSO	^13^C exp. DMSO-*d*_6_	^13^C exp. CPMAS

4	7.80	7.77	7.64^a^	121.81	119.62	119.2	119.1
5	7.23	7.36	7.19^a^	121.55	121.73	121.3	121.2
6	7.20	7.36	7.25^a^	122.32	122.53	122.1	123.2
7	7.24	7.59	7.54^a^	106.50	109.36	110.1	110.7
3a	–	–	–	146.49	145.60	143.3	143.4
7a	–	–	–	135.58	136.21	134.5	134.7

^a 3^*J*_45_ = 7.89, ^4^*J*_46_ = 1.26, ^5^*J*_47_ = 0.78, ^3^*J*_56_ = 7.14, ^4^*J*_57_ = 1.30, ^3^*J*_67_ = 7.99 Hz.

The assignment of the signals of Table 1, Table 2 and Table 3 was straightforward following these steps: i) A NOESY experiment identifies H7 (7.54 ppm) of **5** by its proximity to the *N*-methyl group; ii) the analysis of the ABCD system of the protons H4, H5, H6 and H7, identifies the remaining protons; iii) a series of 2D experiments assign the CH carbons (HMQC) as well as the quaternary carbons C3a and C7a (HMBC).

**Table 2:** Calculated and experimental ^13^C chemical shifts of the imidazole and substituent parts.

Compound	^1^H calcd gas	^1^H calcd DMSO	^1^H exp. HMPA-*d*_18_	^13^C calcd gas	^13^C calcd DMSO	^13^C exp. HMPA-*d*_18_	^13^C exp. CPMAS

**1**							
2	7.64	7.95	8.21	136.78	140.77	141.6	143.1
**1** tri A							
2	8.01	8.32	8.21	139.05	141.86	141.6	143.1
**1** tri B							
2	7.95	8.24	8.21	138.24	141.16	141.6	143.1

Compound	^1^H calcd gas	^1^H calcd DMSO	^1^H exp. DMSO-*d*_6_	^13^C calcd gas	^13^C calcd DMSO	^13^C exp. DMSO-*d*_6_	^13^C exp. CPMAS

**1**							
2			8.20			141.9	
**2**							
2	–	–	–	146.54	151.70	151.2	153.6
Me	2.41	2.50	2.47	14.37	14.42	14.6	12.6

						^13^C exp HMPA-*d*_18_	

**3**^a^**^,^**^b^							
2	–	–	–	138.96	139.54	140.8 ^2^*J*_CF_ = 39.1	135.2 137.8
CF_3_	–	–	–	123.53	123.96	120.0 ^1^*J*_CF_ = 270.1	116.0
**4**							
2	–	–	–	151.48	155.02	153.5	157.1
CH_2_	4.18	4.22	35.0	38.73	38.42	35.0	36.0
C*ipso*	–	–	–	139.73	139.83	137.7	136.2
C*ortho*	7.30	7.54	7.30	129.32	129.28	128.5	~127
C*meta*	7.38	7.49	7.33	128.76	129.44	128.8	~127
C*para*	7.34	7.38	7.22	126.76	127.15	126.5	124.3
**5**							
2	7.41	7.74	8.16^c^	140.83	144.14	144.5	144.6
NMe	3.62	3.80	3.82^c^	29.78	30.69	30.6	29.7

^a^In HMPA-*d_18_* 273 K; ^b 19^F calc.: −63.35 (gas), −63.84 (DMSO); ^19^F exp.: −62.8 (HMPA-*d*_18_); −61.2 (CPMAS). ^c 4^*J*_HMe_ = 0.42 Hz.

**Table 3:** Calculated and experimental ^15^N chemical shifts of the imidazole part.

Compound	^15^N calcd gas	^15^N calcd DMSO	^15^N exp. HMPA-*d*_18_	^15^N exp. CPMAS

**1**				
N1	−247.63	−240.09	−228.4	−221.8
N3	−125.53	−140.75	−134.0	−143.9
**1** tri A				
N1	−227.89	−222.08	−228.4	−221.8
N3	−142.23	−149.24	−134.0	−143.9
**1** tri B				
N1	−227.80	−221.92	−228.4	−221.8
N3	−142.21	−149.18	−134.0	−143.9

Compound	^15^N calcd gas average	^15^N calcd DMSO average	^15^N exp. DMSO-*d*_6_ average	^15^N exp. CPMAS

**1**				
N1/N3	−186.58	−190.42	^a^	
**2**				
N1	−246.87	−240.39	−230.3	−219.5, −224.2
N3	−127.73	−142.15	−137.9	−146.9

			^15^N exp HMPA-*d*_18_	

**3**^b^				
N1	−247.40	−241.51	−230.4	−225.9
N3	−120.36	−132.07	^a^	−142.8
**4**				
N1	−245.40	−241.11	−230.8	−221.8
N3	−130.39	−141.51	−136.6	−147.2
**5**				
N1	−241.25	−232.11	−235.6	−233.4
N3	−128.38	−143.76	−136.4	−135.4

^a^Not observed; ^b^In HMPA-*d*_18_ 273 K.

**Figure 4:**
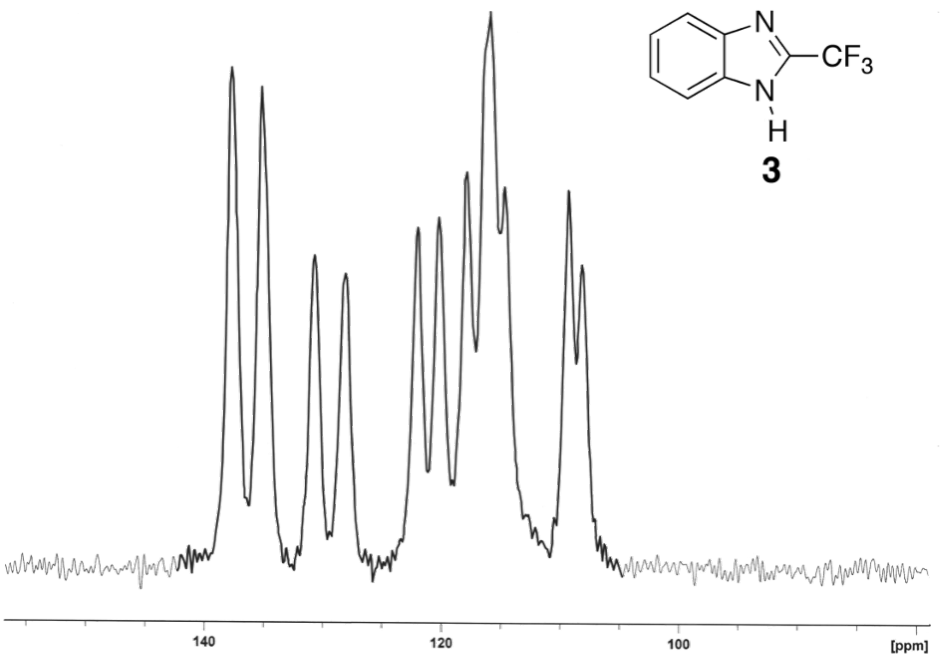
The ^13^C CPMAS NMR spectrum of **3**.

In the case of **3** all signals are considerably split in the solid state (Figure 4).

**Figure 5:**
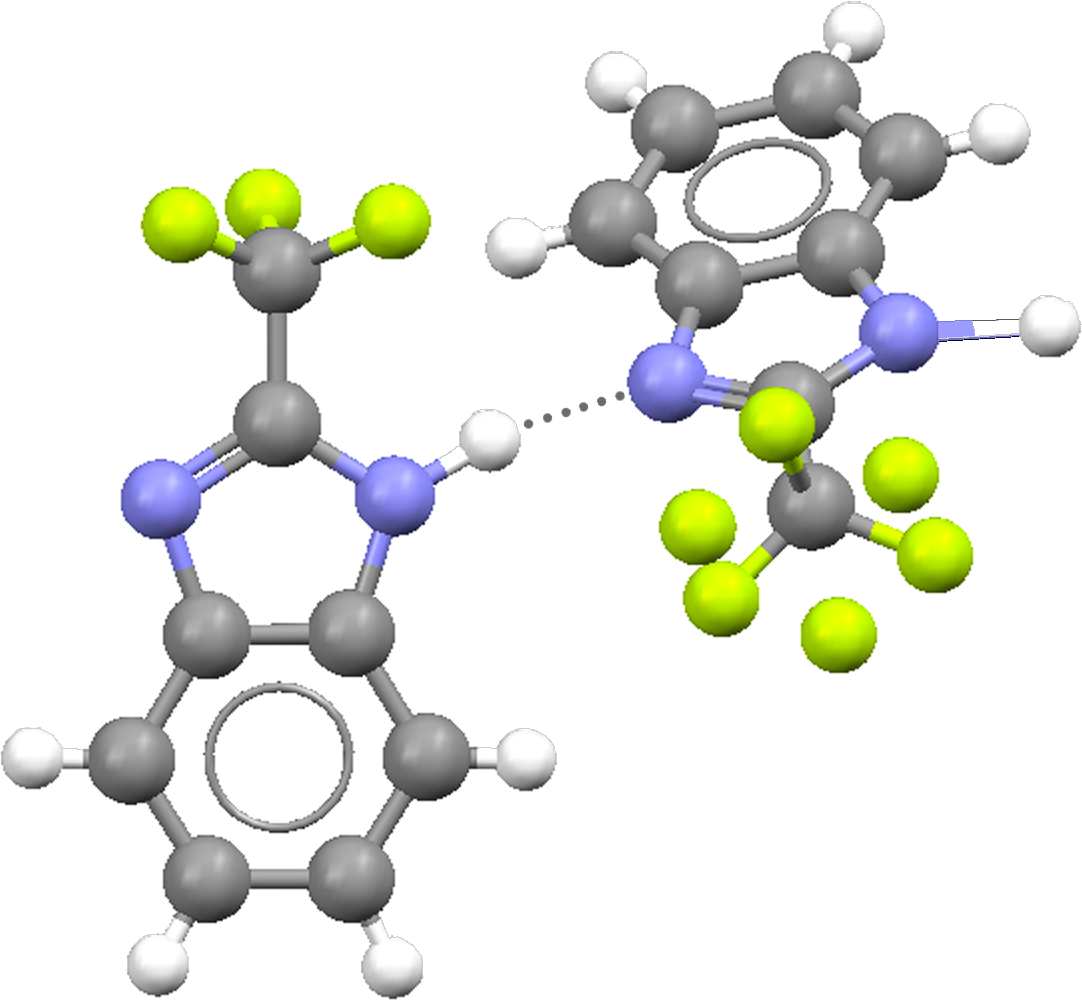
The two independent molecules of **3** drawn with the Mercury program [26].

The X-ray structure of compound **3** is known and reported in the Cambridge Structural Database (refcode: ZAQRIU01) [4,26]. There are two independent molecules and in one of them, the CF_3_ is disordered (Figure 5). The most probable explanation of the splittings of Figure 4 is the existence of two independent molecules, a fact that is well documented in the literature [27–30].

We have collected in Table 4 the different equations obtained from the data of Table 1, Table 2 and Table 3.

**Table 4:** Linear regression equations, intercepts in ppm. If not specified, both trimers yield the same results.

Eq.	No	Intercept	Slope	R^2^	Atom	Exp.	Calcd

1	26	(3.2 ± 0.7)	(0.55 ± 0.10)	0.563	^1^H	solution	gas phase
2	26	(1.2 ± 0.6)	(0.81 ± 0.08)	0.797	^1^H	solution	DMSO
3	59	−(0.2 ± 1.3)	(1.00 ± 0.01)	0.993	^13^C	solution	gas phase
4	59	−(0.7 ± 0.7)	(1.000 ± 0.001)	0.998	^13^C	solution	DMSO
5	59	−(0.7 ± 2.0)	(1.01 ± 0.02)	0.987	^13^C	CPMAS	gas phase
6	59	−(0.8 ± 1.2)	(1.01 ± 0.01)	0.993	^13^C	CPMAS	DMSO
7^a^	58	−(0.2 ± 1.3)	(1.00 ± 0.01)	0.994	^13^C	solution	gas phase
8^a^	58	−(0.7 ± 0.7)	(1.000 ± 0.005)	0.998	^13^C	solution	DMSO
9^a^	53	−(0.6 ± 1.9)	(1.01 ± 0.02)	0.989	^13^C	CPMAS	gas phase
10^a^	53	−(1.1 ± 1.3)	(1.01 ± 0.01)	0.995	^13^C	CPMAS	DMSO
11	6	(6.6 ± 7.2)	(0.95 ± 0.06)	0.986	^13^C	CPMAS	monomer^b^
12	6	(5.2 ± 3.3)	(0.96 ± 0.03)	0.997	^13^C	CPMAS	trimer A^b^
13	6	(3.7 ± 3.4)	(0.98 ± 0.03)	0.997	^13^C	CPMAS	trimer B^b^
14	14	−(22.3 ± 7.3)	(0.86 ± 0.04)	0.978	^15^N	solution	gas
15	14	(10.5 ± 9.1)	(1.02 ± 0.05)	0.976	^15^N	solution	DMSO
16	14	−(51.0 ± 7.1)	(0.72 ± 0.04)	0.970	^15^N	CPMAS	gas
17	14	−(23.5 ± 7.5)	(0.86 ± 0.04)	0.976	^15^N	CPMAS	DMSO
18	10	−(1.4 ± 6.6)	(0.96 ± 0.03)	0.991	^15^N	solution	monomer^b^
19	10	(6.6 ± 10.4)	(1.00 ± 0.05)	0.979	^15^N	solution	trimer^b^
20	10	−(29.8 ± 5.3)	(0.82 ± 0.03)	0.991	^15^N	CPMAS	monomer^b^
21	10	−(23.8 ± 7.5)	(0.85 ± 0.04)	0.984	^15^N	CPMAS	trimer^b^

^a^The ^13^C signal of the CF_3_ group has been removed. ^b^DMSO calculations.

i) The ^1^H chemical shifts are much more consistent with calculations for DMSO as solvent than with those of isolated molecules (gas phase). For 26 points, the R^2^ coefficient increases from 0.56 (eq. 1) to 0.80 (eq. 2). The use of the monomer or the central part of the trimer has no influence. The worse point is H7 of **4** that appears at 7.40 ppm and fitted with eq. 2 has a value of 7.14 ppm. The origin of this discrepancy is that the theoretical conformation corresponding to the X-ray structure is not stable and reverts to the minimum one, which has the benzyl group rotated.

ii) The ^13^C chemical shifts (eqs. 3–13) are very well reproduced by the calculations: high R^2^ values, small intercepts (in several cases, not significant) and slopes close to 1. Systematically, the worse point was the carbon atom of the CF_3_ substituent (halogen substituents produce effects that are not well reproduced by our calculations that not include relativistic corrections) [15], removing it does not significantly modify the regression values (compare eqs. 3–6 with eqs. 7–10). CPMAS values agree better with calculations including DMSO solvent effect (compare eqs. 9 and 10) and also better with trimer B (in turn, slightly better than with trimer A, compare intercept and slopes of eqs. 12 and 13) than with the monomer (eq. 11).

iii) Concerning ^15^N NMR (eqs. 14–21), the R^2^ values are lower than with ^13^C NMR. Both for solution and for CPMAS, the gas phase and DMSO calculations are comparable in terms of R^2^ (eqs. 14–17), however, the values of the slopes (the closer to 1, the better) and intercepts (the closer to 0, the better), clearly favored the DMSO calculations. Surprisingly, the monomer appears preferable to the trimer (eqs. 18,19 and 20,21) which is understandable for the solution but not for the solid state. More complex approaches, such as periodic calculations [31], are necessary.

### Influence of the substituent at position 2 on the tautomerization rate

**Figure 6:**
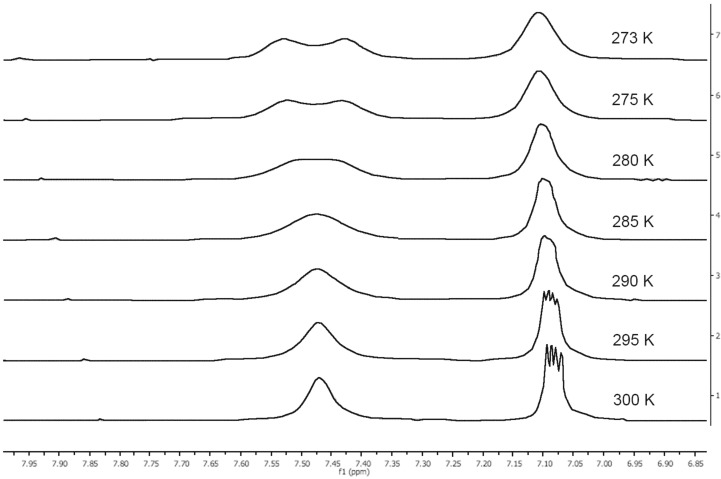
The evolution of the spectrum of **1** with temperature in HMPA-*d*_18_.

We have observed a different behavior for the four NH-benzimidazoles: **1** and **3** yielded average signals in DMSO-*d*_6_ and only in HMPA-*d*_18_ the prototropic exchange was slow, on the other hand **2** and **4** behaved as if the tautomerism was blocked in DMSO-*d*_6_. In the case of **1** a dynamic NMR (DNMR) study in HMPA-*d*_18_ was performed (Figure 6).

The relevant data are: *T*_C_ = 277.5 K and Δ*ν* = 0.12 ppm = 48 Hz; from them and using the Eyring equation [Δ*G*^‡^_TC_ = 19.12 × *T*_C_ × (10.32 + log *T*_C_/*k*_C_)] [32], we obtained: *k*_C_ = 67.88 s^−1^ and Δ*G*^‡^_277.5_ = 58.0 kJ·mol^−1^. This barrier is similar to that of pyrazole in the same solvent (58.6 kJ·mol^−1^ at 289 K) [33].

The effect of the substituent at position 2 on the rate (roughly, CF_3_ and H, fast; CH_2_C_6_H_5_ and CH_3_, slow) is probably the consequence of steric and electronic effects; many years ago, we showed that intramolecular hydrogen bonds also affect the rate [34]. The calculated electrostatic potential minima associated to the lone pair of the N3 follow the tautomerization rate ranking of the molecules (−0.085 au, CF_3_, −0.103, H, −0.104, CH_2_C_6_H_5_ and −0.105, CH_3_).

## Conclusion

The data reported here for NH-benzimidazoles when the prototropy is blocked should be useful to determine the tautomeric composition when there are substituents at positions 4(7) or 5(6), for instance, in the case of omeprazole, a 5(6)-methoxy-1*H*-benzimidazole derivative [35–36]. Besides, solid state results as well as GIAO calculations provide new data to characterize this important family of compounds. Finally, by means of DNMR experiments it was possible to determine the barrier to proton transfer of benzimidazole itself in HMPA-*d*_18_, thus providing a missing value in heterocyclic tautomerism of azoles and benzazoles [33].

## Experimental

Four of the compounds reported in this paper are commercial (Sigma-Aldrich): **1**, **2**, **3** and **5**. We reported the synthesis of the fifth one, **4**, in [37].

### NMR spectroscopy

Solution NMR spectra were recorded on a Bruker DRX 400 (9.4 Tesla, 400.13 MHz for ^1^H, 100.62 MHz for ^13^C and 40.54 MHz for ^15^N) spectrometer with a 5 mm inverse-detection H-X probe equipped with a z-gradient coil, at 300 K. Chemical shifts (δ) are given from internal solvent, DMSO-*d*_6_ 2.49 for ^1^H and 39.5 for ^13^C. Typical parameters for ^1^H NMR spectra were spectral width 4800 Hz and pulse width 8.3 μs at an attenuation level of 0 dB. Typical parameters for ^13^C NMR spectra were spectral width 21 kHz, pulse width 12.5 μs at an attenuation level of −6 dB and relaxation delay 2s, WALTZ-16 was used for broadband proton decoupling; the FIDS were multiplied by an exponential weighting (lb = 1Hz) before Fourier transformation.

Inverse proton detected heteronuclear shift correlation spectra, (^1^H,^13^C) gs-HMQC and (^1^H,^13^C) gs-HMBC, were acquired and processed using standard Bruker NMR software and in nonphase-sensitive mode. Gradient selection was achieved through a 5% sine truncated shaped pulse gradient of 1 ms.

Selected parameters for (^1^H,^13^C) gs-HMQC and (^1^H,^13^C) gs-HMBC spectra were spectral width 4800 Hz for ^1^H and 20.5 kHz for ^13^C, 1024 × 256 data set, number of scans 2 (gs-HMQC) or 4 (gs-HMBC) and relaxation delay 1s. The FIDs were processed using zero filling in the *F_1_* domain and a sine-bell window function in both dimensions was applied prior to Fourier transformation. In the gs-HMQC experiments, GARP modulation of ^13^C was used for decoupling.

Selected parameters for (^1^H,^15^N) gs-HMQC and (^1^H,^15^N) gs-HMBC spectra were spectral width 3500 Hz for ^1^H and 12.5 kHz for ^15^N, 1024 × 256 data set, number of scans 4, relaxation delay 1s, 37–60 ms delay for evolution of the ^15^N,^1^H long-range coupling. The FIDs were processed using zero filling in the *F_1_* domain and a sine-bell window function in both dimensions was applied prior to Fourier transformation.

For ^19^F NMR (379.50 MHz) a 5 mm QNP direct-detection probehead equipped with a z-gradient coil, at 300 K was required.

Chemical shifts (δ) are given from internal solvent, DMSO-*d*_6_ 2.49 and HMPA-*d*_18_ 2.57 for ^1^H; 39.5 and 35.8 for ^13^C, and for ^15^N and ^19^F NMR, nitromethane (0.00) and one drop of CFCl_3_ in CDCl_3_ (0.00) were used as external references

Typical parameters for ^1^H NMR were: spectral width 4800 Hz and pulse width 10.25 μs at an attenuation level of −3.0 dB. For ^13^C NMR: spectral width 21 kHz, pulse width 8.75 μs at an attenuation level of −3 dB and relaxation delay 2 s, WALTZ-16 was used for broadband proton decoupling; the FIDS were multiplied by an exponential weighting (lb = 1Hz) before Fourier transformation. For ^19^F NMR: spectral width 55 kHz, pulse width 13.57 μs at an attenuation level of −6 dB and relaxation delay 1s.

Variable temperature: A Bruker BVT3000 temperature unit was used to control the temperature of the cooling gas stream and an exchanger to achive low temperatures. To avoid problems at low temperatures caused by air moisture, pure nitrogen was used as bearing, driving and cooling gas. When not stated explicitly, the temperature was 293 K.

We have always used a 1.4 M solution of the compounds either in DMSO-*d*_6_ or in HMPA-*d*_18_; this corresponds, in the case of **1**, to 10 mg in 0.6 mL. To avoid water contamination, a new sealed ampoule was open for each experiment.

Solid state ^13^C (100.73 MHz) and ^15^N (40.60 MHz) CPMAS NMR spectra were obtained on a Bruker WB 400 spectrometer at 300 K using a 4 mm DVT probehead. Samples were carefully packed in 4 mm diameter cylindrical zirconia rotors with Kel-F end-caps. Operating conditions involved 2.9 µs 90° ^1^H pulses and decoupling field strength of 86.2 kHz by TPPM sequence. ^13^C spectra were originally referenced to a glycine sample and then the chemical shifts were recalculated to the Me_4_Si [for the carbonyl atom δ (glycine) = 176.1] and ^15^N spectra to ^15^NH_4_Cl and then converted to nitromethane scale using the relationship: δ ^15^N(nitromethane) = δ^1 5^N(ammonium chloride) − 338.1.

Typical acquisition parameters for ^13^C CPMAS were: spectral width, 40 kHz; recycle delay, 5–60 s; acquisition time, 30 ms; contact time, 2–4 ms; and spin rate, 12 kHz. In order to distinguish protonated and non-protonated carbon atoms, the NQS (Non-Quaternary Suppression) experiment by conventional cross-polarization was recorded; before the acquisition the decoupler is switched off for a very short time of 25 μs [38–40]. For ^15^N CPMAS were: spectral width, 40 kHz; recycle delay, 5–60 s; acquisition time, 35 ms; contact time, 7–9 ms; and spin rate, 6 kHz.

Solid state ^19^F (376.94 MHz) NMR spectra were obtained on a Bruker WB 400 spectrometer using a MAS DVT BL2.5 X/F/H probe. Samples were carefully packed in 2.5 mm diameter cylindrical zirconia rotors with Kel-F end-caps.

Typical acquisition parameters ^19^F{^1^H} MAS were: spectral width, 75 kHz; recycle delay, 10 s; pulse width, 2.5 μs and proton decoupling field strength of 100 kHz by SPINAL-64 sequence; recycle delay, 10 s; acquisition time, 25 ms; 128 scans; and spin rate, 25 kHz. The ^19^F spectra was referenced to ammonium trifluoroacetate sample and then the chemical shifts were recalculated to the CFCl_3_ [δ (CF_3_CO_2_NH_4_^+^) = −72.0].

### Computational details

Using the Gaussian 09 facilities [41], GIAO [42–43]/B3LYP [44–46]/6-311++G(d,p) [47–48] calculations were carried out; DMSO effects were calculated using the PCM continuum model [49] also with the Gaussian 09 series of programs.

## Supporting Information

File 1 Optimized geometry of the systems, and chemical shifts in gas phase and PCM/DMSO environment.
