# Supplementary material for: An experimental and theoretical NMR study of NH-benzimidazoles in solution and in the solid state: proton transfer and tautomerism
Source: Beilstein J Org Chem. 2014 Jul 16;10:1620–9. doi: 10.3762/bjoc.10.168 (PMC4142846; doi:10.3762/bjoc.10.168)
Supplement: File 1 — Optimized geometry of the systems, and chemical shifts in gas phase and PCM/DMSO environment. [file Beilstein_J_Org_Chem-10-1620-s001.pdf]

Supporting Information
for

An experimental and theoretical NMR study of NH-benzimidazoles in solution and in the solid state: proton transfer and tautomerism

Carla I. Nieto[1], Pilar Cabildo[1], M. Ángeles García[1], Rosa M. Claramunt*[1], Ibon Alkorta*[2] and José Elguero[2]

Address: [1]Departamento de Química Orgánica y Bio-Orgánica, Facultad de Ciencias, UNED Senda del Rey 9, E-28040 Madrid, Spain and [2]Instituto de Química Médica, CSIC, Juan de la Cierva, 3, E-28006 Madrid, Spain

Email: Rosa M. Claramunt* - rclaramunt@ccia.uned.es; Ibon Alkorta* - ibon@iqm.csic.es

*Corresponding author

Optimized geometry of the systems, and chemical shifts in gas phase and PCM/DMSO environment

Cartesian coordinates and GIAO results of the systems studied.

Cartesian Coordinates, Å	GIAO Results (gas phase), ppm.			GIAO results (DMSO), ppm		
**Bzml**	Atom	Abs.	Rel.	Atom	Abs.	Rel.
N,1.0447718408,1.5952001503,0.	1N	101.09	-247.63	1N	93.12	-240.09
C,-0.1269434872,2.3178825537,0.	2C	40.41	136.78	2C	36.27	140.77
N,-1.1973208861,1.5720869895,0.	3N	-27.99	-125.53	3N	-11.90	-140.75
C,0.6897292343,0.2567343558,0.	4C	44.65	132.70	4C	43.78	133.54
C,-0.7239690703,0.2658149217,0.	5C	32.22	144.67	5C	32.69	144.22
C,-1.4271684315,-0.9434622051,0.	6C	56.11	121.67	6C	58.28	119.58
C,-0.6952548717,-2.1234790872,0.	7C	56.10	121.68	7C	55.98	121.79
C,0.7131279626,-2.1140327959,0.	8C	54.85	122.88	8C	54.68	123.04
C,1.4320051919,-0.9241180748,0.	9C	70.07	108.23	9C	67.20	110.99
H,-2.5104902505,-0.9450248201,0.	10H	23.91	7.81	10H	23.93	7.79
H,-1.2144606297,-3.0749736326,0.	11H	24.53	7.20	11H	24.40	7.34
H,1.2487936774,-3.0563873731,0.	12H	24.50	7.24	12H	24.32	7.41
H,1.9784271004,1.9730078446,0.	13H	23.75	7.96	13H	22.80	8.88
H,2.5160257678,-0.9226868796,0.	14H	24.41	7.32	14H	24.05	7.67
H,-0.1206469281,3.3985992527,0.	15H	24.08	7.64	15H	23.77	7.95
**2MeBzml**	Atom	Abs.	Rel.	Atom	Abs.	Rel.
N,1.0786363707,1.1170915268,0.	1N	100.29	-246.87	1N	93.44	-240.39
C,-0.0786777599,1.8738466247,0.	2C	30.28	146.54	2C	24.92	151.70
N,-1.1576925089,1.1338766506,0.	3N	-25.65	-127.73	3N	-10.41	-142.15
C,0.701913,-0.2163545654,0.	4C	41.71	135.53	4C	40.97	136.25
C,-0.710475032,-0.1816292591,0.	5C	31.80	145.08	5C	32.29	144.60
C,-1.4344376075,-1.377124155,0.	6C	57.03	120.78	6C	59.36	118.54
C,-0.724212985,-2.5721869176,0.	7C	56.57	121.22	7C	56.70	121.09
C,0.6825192319,-2.5883626677,0.	8C	55.86	121.91	8C	55.91	121.86
C,1.4221254757,-1.4091297328,0.	9C	70.51	107.79	9C	67.90	110.32
H,-2.5177659031,-1.3597234831,0.	10H	24.02	7.70	10H	24.04	7.68
H,-1.2615091178,-3.5136574743,0.	11H	24.63	7.11	11H	24.50	7.23
H,1.2021976605,-3.5396509248,0.	12H	24.68	7.06	12H	24.52	7.21
H,2.0203519125,1.474464536,0.	13H	24.17	7.56	13H	23.29	8.41
H,2.5061581923,-1.4268879489,0.	14H	24.53	7.21	14H	24.19	7.54
C,-0.0490998154,3.3653337307,0.	15C	167.53	14.37	15C	167.48	14.42
H,0.4641704726,3.7553876066,0.8848570963	16H	29.57	2.31	16H	29.32	2.56
H,0.4641704726,3.7553876066,-0.8848570963	17H	29.57	2.31	17H	29.32	2.56
H,-1.0728564891,3.7364282664,0.	18H	29.28	2.60	18H	29.49	2.39
**2CF3Bzlm**	Atom	Abs.	Rel.	Atom	Abs.	Rel.
N,-1.088545208,0.1282749352,0.	1N	100.85	-247.40	1N	94.62	-241.51
C,0.0704393989,0.8663745508,0.	2C	38.15	138.96	2C	37.55	139.54
N,1.1573628356,0.1495772606,0.	3N	-33.45	-120.36	3N	-21.06	-132.07
	4C	45.10	132.27	4C	44.09	133.24

C,-0.7022419804,-1.199046167,0.	5C	32.35	144.54	5C	33.05	143.87
C,0.712070524,-1.1625164605,0.	6C	55.09	122.65	6C	56.92	120.89
C,1.4430587625,-2.3567601223,0.	7C	53.60	124.08	7C	52.81	124.84
C,0.7343073058,-3.5482197699,0.	8C	51.73	125.89	8C	50.90	126.68
C,-0.6757631543,-3.5668563621,0.	9C	69.19	109.07	9C	66.15	112.00
C,-1.4212274846,-2.3956953916,0.	10H	23.32	8.38	10H	22.55	9.13
H,-2.0259472781,0.4991465566,0.	11C	54.18	123.53	11C	53.72	123.96
C,0.0402828589,2.3688693244,0.	12F	228.54	-57.07	12F	228.53	-57.06
F,0.639338844,2.8897334643,1.0834580854	13F	248.18	-75.90	13F	249.74	-77.40
F,-1.2489897468,2.8038254873,0.	14F	228.54	-57.07	14F	228.53	-57.06
F,0.639338844,2.8897334643,-1.0834580854	15H	23.84	7.87	15H	23.83	7.88
H,2.5259765006,-2.3350551494,0.	16H	24.30	7.43	16H	24.14	7.59
H,1.2713771803,-4.4896811484,0.	17H	24.36	7.37	17H	24.16	7.57
H,-1.1903439433,-4.5209024332,0.	18H	24.42	7.31	18H	24.06	7.67
H,-2.5047081294,-2.4162405591,0.						
**2BnBzml**	Atom	Abs.	Rel.	Atom	Abs.	Rel.
N,0.9468942508,1.5351059604,0.3745382542	1N	98.73	-245.40	1N	94.20	-241.11
C,-0.173709052,2.2852340815,0.0831540611	2C	25.16	151.48	2C	21.47	155.02
N,-1.1978799082,1.5480768326,-0.2649260888	3N	-22.84	-130.39	3N	-11.09	-141.51
C,0.6047063981,0.2037369981,0.2087849278	4C	43.02	134.27	4C	41.81	135.43
C,-0.7475948692,0.2355927341,-0.2018860808	5C	32.78	144.14	5C	33.51	143.43
C,-1.4223041431,-0.9599942499,-0.4656572137	6C	57.36	120.46	6C	59.39	118.51
C,-0.7250143526,-2.1520977471,-0.3092653792	7C	55.86	121.91	7C	55.54	122.22
C,0.6204851861,-2.1658238024,0.1028100195	8C	55.10	122.64	8C	54.94	122.79
C,1.3104137955,-0.9872689073,0.370070241	9C	71.09	107.24	9C	68.45	109.79
H,-2.458516213,-0.9446413836,-0.7818342019	10H	23.98	7.74	10H	24.02	7.70
H,-1.224405677,-3.0935703945,-0.5075897416	11H	24.46	7.27	11H	24.35	7.38
H,1.1312711954,-3.1154571142,0.2140294436	12H	24.62	7.12	12H	24.45	7.28
H,1.814355598,1.8995278248,0.7361789435	13H	24.52	7.22	13H	23.54	8.16
H,2.3471063055,-1.0030357793,0.6867037224	14H	24.79	6.95	14H	24.41	7.32
C,-0.1626925734,3.7869566319,0.1223905701	15C	142.23	38.73	15C	142.55	38.42
H,0.2429918958,4.1619673883,-0.8241501432	16H	27.96	3.88	16H	27.78	4.06
H,-1.2061247413,4.1064323338,0.1632462562	17H	27.33	4.49	17H	27.44	4.38
C,0.6302454415,4.3693279814,1.2773032201	18C	37.35	139.73	18C	37.25	139.83
C,1.858361622,5.0010687593,1.0608452229	19C	48.87	128.64	19C	48.18	129.31
C,0.1493760059,4.2716387772,2.5892685522	20C	47.45	130.00	20C	48.25	129.24
C,2.5931603639,5.5220371137,2.1274547165	21C	48.98	128.53	21C	48.13	129.35
H,2.2398689989,5.0959653623,0.0487782545	22H	24.43	7.30	22H	24.09	7.63
C,0.8784210142,4.7919830467,3.6546609616	23C	48.29	129.20	23C	47.93	129.54
H,-0.802685091,3.7843423418,2.7735441851	24H	24.43	7.31	24H	24.28	7.45
C,2.1047522459,5.4183732233,3.4270673779	25C	50.82	126.76	25C	50.41	127.15
H,3.5417290324,6.0126795613,1.9395430865	26H	24.34	7.39	26H	24.14	7.58
	27H	24.37	7.36	27H	24.33	7.40

H,0.4889600395,4.7120562898,4.6634705167<br>H,2.6711494214,5.8253642461,4.2569768556	28H	24.39	7.34	28H	24.35	7.38
**NMeBzml**	Atom	Abs.	Rel.	Atom	Abs.	Rel.
N,0.1973531317,1.5292476812,0.	1N	94.34	-241.25	1N	84.68	-232.11
C,-1.1613902791,1.7524415488,0.	2C	36.21	140.83	2C	32.77	144.14
N,-1.8822503836,0.6633289358,0.	3N	-24.97	-128.38	3N	-8.71	-143.76
C,0.3575600246,0.1542582597,0.	4C	41.66	135.58	4C	41.00	136.21
C,-0.9567376675,-0.3699383414,0.	5C	30.34	146.49	5C	31.26	145.60
C,-1.152783442,-1.7545046265,0.	6C	55.96	121.81	6C	58.24	119.62
C,-0.0303929082,-2.5731192871,0.	7C	56.23	121.55	7C	56.05	121.73
C,1.2710521359,-2.0353519288,0.	8C	55.43	122.32	8C	55.21	122.53
C,1.4897519541,-0.6618042503,0.	9C	71.86	106.50	9C	68.88	109.36
H,-2.155902354,-2.1639329707,0.	10H	23.92	7.80	10H	23.95	7.77
H,-0.1541163199,-3.6500276907,0.	11H	24.50	7.23	11H	24.37	7.36
H,2.1216920507,-2.7072867887,0.	12H	24.54	7.20	12H	24.37	7.36
H,2.4944208999,-0.2548272784,0.	13H	24.50	7.24	13H	24.13	7.59
H,-1.5560175517,2.759529197,0.	14H	24.32	7.41	14H	23.98	7.74
C,1.2507075923,2.5262539525,0.	15C	151.53	29.78	15C	150.58	30.69
H,1.8784428489,2.427710146,0.8900347714	16H	28.22	3.62	16H	28.09	3.75
H,1.8784428489,2.427710146,-0.8900347714	17H	28.22	3.62	17H	28.09	3.75
H,0.7995486789,3.5188423855,0.	18H	28.21	3.63	18H	27.95	3.89

Cartesian Coordinates, Å	GIAO Results (gas phase), ppm.			GIAO results (DMSO), ppm		
**(Bzml)3-A**	Atom	Abs.	Rel.	Atom	Abs.	Rel.
N,3.8335149515,-0.3241064127,-0.8698501156	1N	80.36	-228.02	1N	74.29	-222.28
C,4.3438343002,-0.527635567,-2.1247213037	2C	37.37	139.71	2C	37.17	139.91
N,5.5237325073,-1.0982372018,-2.1388159035	3N	-23.47	-129.80	3N	-8.92	-143.57
C,4.7749611457,-0.812826972,0.0142079066	4C	42.57	134.70	4C	42.38	134.88
C,5.8294959424,-1.2944663957,-0.8000560101	5C	31.13	145.72	5C	32.05	144.84
C,6.9623273776,-1.8647393268,-0.2082829153	6C	57.14	120.68	6C	58.85	119.03
C,7.0097972074,-1.9379462942,1.1779679098	7C	57.86	119.98	7C	56.76	121.04
C,5.9532042134,-1.4536477932,1.9747387759	8C	56.94	120.87	8C	55.80	121.97
C,4.8191533745,-0.8829109185,1.4081990381	9C	69.06	109.20	9C	66.69	111.48
H,7.7731910996,-2.2347229669,-0.8248156977	10H	23.97	7.75	10H	23.88	7.84
H,7.8764868694,-2.3746909849,1.6615790209	11H	24.62	7.12	11H	24.39	7.34
H,6.0282153687,-1.526073047,3.0541261966	12H	24.67	7.07	12H	24.39	7.35
H,2.9185260403,0.0900256637,-0.6432723922	13H	17.55	13.98	13H	15.87	15.61
H,4.00942387,-0.5075624991,2.0233323133	14H	24.36	7.37	14H	23.93	7.79
H,3.7940001449,-0.22943233,-3.0064267181	15H	23.84	7.87	15H	23.61	8.10
C,0.6867726046,2.0383102178,-0.039294426	16C	33.07	143.85	16C	33.32	143.62
C,0.1088435139,-0.0270019873,-0.0821014673	17C	38.06	139.05	17C	35.14	141.86
C,-0.6958806427,1.9952320887,0.2528188497	18C	43.17	134.13	18C	42.51	134.76
C,1.3574790837,3.265647384,-0.0829542826	19C	57.99	119.85	19C	59.63	118.28
H,0.1196270345,-1.1038948443,-0.1713360067	20H	23.70	8.01	20H	23.38	8.32
C,-1.4393884449,3.1498843646,0.5043480542	21C	68.22	110.00	21C	66.28	111.88
C,0.6215055654,4.4159188404,0.1670676116	22C	55.66	122.10	22C	55.85	121.91
H,2.4171232624,3.3089633525,-0.3051746868	23H	23.82	7.89	23H	23.79	7.93
C,-0.7563834253,4.3588555228,0.4562769718	24C	54.80	122.93	24C	55.01	122.73
H,-2.4992466709,3.1086961945,0.7262384178	25H	24.24	7.49	25H	23.95	7.77
H,1.114122929,5.3810447083,0.1400567542	26H	24.47	7.26	26H	24.34	7.39
H,-1.2952102305,5.2803324304,0.6450511472	27H	24.45	7.28	27H	24.34	7.39
N,1.1587205699,0.7473044041,-0.2441409922	28N	-10.33	-142.23	28N	-2.92	-149.24
N,-1.0330307843,0.6540653638,0.2164861453	29N	80.22	-227.89	29N	74.08	-222.08
C,-4.7074192737,-0.824275509,-0.109898448	30C	34.43	142.54	30C	34.36	142.61
C,-4.0220079739,-1.0947327594,1.9096362952	31C	39.44	137.72	31C	35.56	141.46
C,-5.7511453891,-1.4808499157,0.5764598996	32C	44.57	132.78	32C	43.47	133.84
C,-4.8521987227,-0.5034115691,-1.4636968556	33C	57.56	120.27	33C	59.03	118.86
H,-3.4319833415,-1.0900585896,2.8145937633	34H	23.72	7.99	34H	23.37	8.33
C,-6.9488598641,-1.8349572782,-0.0438912437	35C	68.61	109.63	35C	66.37	111.78
C,-6.0416523086,-0.8531083713,-2.0874674093	36C	53.97	123.73	36C	55.00	122.73
H,-4.0584097316,0.0011689761,-2.0011460585	37H	23.79	7.92	37H	23.79	7.92
H,-5.7576531346,-2.0719453472,2.6419337827	38H	23.57	8.14	38H	22.74	8.94
C,-7.0738503753,-1.5086916277,-1.3885211084	39C	52.83	124.83	39C	53.83	123.87

H,-7.7457576772,-2.3395707188,0.4896023196	40H	24.24	7.49	40H	23.93	7.79
H,-6.1832301906,-0.6181497701,-3.1357422954	41H	24.30	7.43	41H	24.28	7.45
H,-7.9872038121,-1.7648049195,-1.9124028021	42H	24.28	7.45	42H	24.21	7.52
N,-3.6421992565,-0.5998628747,0.7580891321	43N	-12.94	-139.76	43N	-5.34	-146.95
N,-5.2767443531,-1.6382322993,1.869928173	44N	98.38	-245.06	44N	92.22	-239.24
H,-1.9580363731,0.2358605791,0.3895996602	45H	17.33	14.19	45H	16.05	15.43
**(Bzml)3-B**	Atom	Abs.	Rel.	Atom	Abs.	Rel.
N,0,3.8117500763,-0.3785196023,-0.8506143272	1N	81.17	-228.79	1N	74.06	-222.06
C,0,4.2271544252,-0.6024871592,-2.1365764163	2C	38.34	138.78	2C	35.22	141.78
N,0,5.4196905998,-1.1381874192,-2.2305336586	3N	-23.28	-129.98	3N	-9.23	-143.27
C,0,4.8356651524,-0.813210648,-0.032405302	4C	42.31	134.95	4C	41.96	135.29
C,0,5.8368801919,-1.2866634431,-0.9158531949	5C	31.24	145.62	5C	32.20	144.69
C,0,7.0306524937,-1.8063424402,-0.402479416	6C	57.35	120.48	6C	59.13	118.76
C,0,7.1905882071,-1.8385215442,0.9768520229	7C	57.46	120.36	7C	56.45	121.34
C,0,6.1858470463,-1.3630646063,1.8429971884	8C	56.95	120.86	8C	56.04	121.74
C,0,4.9927880345,-0.8424116754,1.3548625901	9C	69.06	109.20	9C	67.27	110.92
H,0,7.8010209765,-2.1698833298,-1.072471531	10H	23.93	7.78	10H	23.89	7.82
H,0,8.1062878182,-2.2357282864,1.400512215	11H	24.51	7.23	11H	24.28	7.45
H,0,6.3484425689,-1.4027909073,2.9143921956	12H	24.62	7.12	12H	24.38	7.35
H,0,2.9057874924,0.0189695985,-0.5655595624	13H	17.22	14.30	13H	15.86	15.62
H,0,4.2228445134,-0.4748841511,2.023479897	14H	24.32	7.41	14H	24.06	7.66
H,0,3.5999433153,-0.3454892286,-2.9787121114	15H	23.89	7.83	15H	23.61	8.10
C,0,0.7077819744,1.9799113199,0.0653539882	16C	33.09	143.84	16C	33.59	143.35
C,0,0.1043147301,-0.0782976734,0.0947328422	17C	38.90	138.24	17C	35.86	141.16
C,0,-0.6711141291,1.9635848884,0.3767744798	18C	43.26	134.04	18C	42.65	134.62
C,0,1.3932475851,3.1964595524,-0.0257204236	19C	57.85	119.99	19C	59.48	118.42
H,0,0.1009624837,-1.1575880195,0.0412033422	20H	23.77	7.95	20H	23.47	8.24
C,0,-1.3963651868,3.1350298181,0.6028742856	21C	68.04	110.18	21C	66.15	112.00
C,0,0.6751970164,4.3633801094,0.1977975999	22C	55.74	122.02	22C	55.89	121.87
H,0,2.4503152625,3.2191270412,-0.2628020026	23H	23.81	7.91	23H	23.82	7.90
C,0,-0.6990430187,4.3330086793,0.5074729409	24C	55.02	122.72	24C	55.06	122.68
H,0,-2.4528052049,3.1143817413,0.8431314221	25H	24.24	7.48	25H	23.97	7.75
H,0,1.1794642335,5.3207508893,0.1345117992	26H	24.46	7.28	26H	24.35	7.38
H,0,-1.2233195341,5.2666911831,0.6762226613	27H	24.48	7.26	27H	24.34	7.39
N,0,1.161166954,0.6773358625,-0.1054458847	28N	-10.35	-142.21	28N	-2.99	-149.18
N,0,-1.0249227786,0.6262186389,0.3873132876	29N	80.13	-227.80	29N	73.91	-221.92
C,0,-4.2450868003,-0.9503955797,2.0800056724	30C	34.64	142.35	30C	34.39	142.58
C,0,-4.5552530998,-0.8908532166,-0.046376941	31C	40.10	137.08	31C	36.09	140.94
C,0,-5.5191819624,-1.5125330608,1.851314911	32C	44.69	132.66	32C	43.58	133.73
C,0,-3.748857369,-0.8460551913,3.3836606867	33C	57.72	120.12	33C	59.27	118.62
H,0,-4.4351673939,-0.7342871745,-1.108501074	34H	23.80	7.91	34H	23.43	8.27
C,0,-6.3275197579,-1.9811367152,2.8866021322	35C	68.72	109.52	35C	66.40	111.76
	36C	53.87	123.82	36C	54.96	122.77

C,0,-4.5480713977,-1.3106346523,4.4187682753	37H	23.77	7.94	37H	23.78	7.94
H,0,-2.7725773875,-0.4158844615,3.5717354186	38H	23.57	8.14	38H	22.78	8.91
H,0,-6.4845010058,-1.7750345558,-	39C	52.79	124.86	39C	53.68	124.01
0.0499684377	40H	24.26	7.47	40H	23.95	7.77
C,0,-5.8175657301,-1.8694601798,4.1740829655	41H	24.26	7.47	41H	24.24	7.49
H,0,-7.3047962453,-2.4120535747,2.7035021526	42H	24.20	7.53	42H	24.12	7.61
H,0,-4.1909515883,-1.2437830112,5.4396126303	43N	-12.44	-140.24	43N	-4.88	-147.39
H,0,-6.4104317334,-2.2208117357,5.0103471361	44N	98.50	-245.18	44N	92.32	-239.34
N,0,-3.672987563,-0.5734570572,0.8679505625	45H	17.31	14.21	45H	16.05	15.43
N,0,-5.6853996334,-1.4580147914,0.4756950378						
H,0,-1.9558126315,0.2258885091,0.5703557448						
